# Relationship Between Prehospital Time and 24-h Mortality in Road Traffic-Injured Patients in Laos

**DOI:** 10.1007/s00268-022-06445-9

**Published:** 2022-01-18

**Authors:** Takaaki Suzuki, Oulaivanh Phonesavanh, Snong Thongsna, Yoshiaki Inoue, Masao Ichikawa

**Affiliations:** 1 Department of Global Public Health, Faculty of Medicine, University of Tsukuba, 1-1-1 Tennodai, Tsukuba, Ibaraki 305-8575 Japan; 2 Department of Emergency and Critical Care Medicine, Faculty of Medicine, University of Tsukuba, 1-1-1 Tennodai, Tsukuba, Ibaraki 305-8575 Japan; 3 Emergency Department, Mittaphab Hospital, Ministry of Health, Vientiane, Lao People’s Democratic Republic; 4 Ministry of Health, Vientiane, Lao People’s Democratic Republic

## Abstract

**Background:**

Road traffic injury has long been regarded as a “time-dependent disease.” However, shortening the prehospital time might not improve the outcome in developing countries given the current quality of in-hospital care. We aimed to examine the relationship between the prehospital time and 24-h mortality among road traffic victims in Laos.

**Methods:**

A prospective observational study was conducted using the trauma registry data on traffic-injured patients who were transported by ambulance to a trauma center in the capital city of Laos from May 2018 to April 2019. The analysis focused on patients with non-mild conditions, whose outcomes could be affected by the prehospital time. To examine the relationship between a prehospital time of <60 min and 24-h mortality, a generalized estimating equation model was used incorporating the inverse probability weights utilizing the propensity score for the prehospital time.

**Results:**

Of 701 patients, 73% were men, 91% were riding 2- or 3-wheel motor vehicles during the crash, and 68% had a prehospital time of <60 min. A total of 35 patients died within 24 h after the crash. Compared with those who survived, individuals who died tended to have head and torso injuries. The proportions of 24-h mortality were 4.7% and 5.4% in patients whose prehospital time was <60 min and ≥60 min, respectively. No significant relationship was found between the prehospital time and 24-h mortality.

**Conclusion:**

A shorter prehospital time was not associated with the 24-h survival among road traffic victims in Laos.

**Supplementary Information:**

The online version contains supplementary material available at 10.1007/s00268-022-06445-9.

## Introduction

Road traffic injuries have long been regarded as a “time-dependent disease.” Hospital arrival within an hour after the injury (the so-called “golden hour”) can improve the patient’s health outcomes [1–9]. Currently, minimizing the time interval between traffic crashes and the provision of first professional emergency care is one of the global road safety performance targets [10]. To achieve this target, an emergency medical service (EMS) system must be developed. However, many developing countries lack EMS systems, and traffic deaths prior to hospital arrival are partly attributable to the lack of timely post-crash responses and access to hospitals [11–14].

The World Health Organization advocates the establishment of EMS systems in developing countries [12]; however, shortening of the prehospital time might not improve the outcome given the current quality of in-hospital care. In developing countries, the hospital facilities may not be fully equipped; additionally, the healthcare workers may not have the necessary skills to resuscitate severely injured patients with head injuries and torso injuries with major bleeding [14–18], which are two major causes of trauma-related death in the early phase. Therefore, the effect of shortening the prehospital time could be less than expected, unless both the quality of in-hospital care and prehospital care is improved.

The relationship between the prehospital time and outcome has been examined mainly in developed countries [1–9, 19–29]; several studies have reported that the shorter the prehospital time, the better the outcome [30, 31]. However, it remains unclear whether this finding is applicable to developing countries, especially in low-resource settings, because of the current quality of in-hospital care.

Conversely, previous studies have two major limitations in estimating the effect of prehospital time on the outcome. First, patients with mild conditions whose outcomes were not affected by the prehospital time were included in the analyses. If the prehospital time of these patients was prolonged because they were not in urgent condition, the impact of the prehospital time might have been underestimated, especially when the patients with mild conditions comprised the majority of the study population. Therefore, it is reasonable to exclude them.

Second, the factors influencing the outcomes, namely vital signs, were inappropriately adjusted in the analyses. Generally, the vital signs measured upon hospital arrival are adjusted in analysis; however, this adjustment might have underestimated the effect of the prehospital time on the outcomes because the possible aggravation of vital signs from the scene to the hospital (due to prehospital time) was cancelled out due to the adjustment. To estimate the effect of the prehospital time on the outcomes, the analysis should control for vital signs measured at the scene.

In Laos, one of the 46 least developed countries, traffic injury is currently one of the main causes of total death [32]. The number of traffic-related deaths per 100,000 people has nearly doubled in the past decade; of the 7.3 million people living in Laos, 1,031 traffic victims died in 2020 [10, 33]. Rescue teams with ambulances have been voluntarily formed by citizens to respond to a rapid increase in traffic victims, and the EMS system has been informally established in the capital city. This situation allowed the examination of whether shortening the prehospital time is beneficial in the context of low-resource settings. Therefore, this study aimed to examine the relationship between a prehospital time of <60 min and the 24-h mortality among traffic victims transported by ambulance in Laos. To overcome the limitations of previous studies, our analysis focused on victims with non-mild conditions, and vital signs at the scene were adjusted.

## Materials and methods

### Study setting and participants

A prospective observational study was conducted in traffic victims transported by ambulance from the scene to Mittaphab Hospital in Vientiane Capital, the capital city of Laos, from May 1, 2018, to April 30, 2019. The patients transported by non-ambulance vehicles were excluded because their condition was mostly mild, and their mortality risk would not be affected by the prehospital time. In fact, nearly all such patients survived. In Laos, this hospital is the only trauma center, and traffic victims are largely motorcycle riders as motorcycles are the primary mode of transportation. To examine the association of prehospital time with mortality, patients transferred from other hospitals were excluded. Patients who experienced cardiac pulmonary arrest (CPA) at the scene were also excluded because they hardly survived and their shock index (SI) could not be calculated, while patients who experienced CPA on the way to the hospital were included.

### EMS in Laos

During the study period, the EMS consisted of 7 rescue teams with assigned emergency call numbers, 34 ambulances, and 650 crew members at Vientiane Capital, which has a population of nearly 0.7 million people and an area of 3920 km^2^. All rescue teams are trained to carry out primary trauma care, such as basic life support, cervical spine protection, manual airway management and pressure hemostasis. Occasionally, the ambulance vehicles without equipment for measuring the vital signs were used to reach the scene, resulting in missing data on vital signs; however, this happened most likely at random because the ambulance vehicles were not systematically dispatched.

Almost all patients with severe trauma were either directly transported from the scene or transferred from other hospitals to Mittaphab Hospital. There were no certified emergency physicians, and the international standard resuscitation was not able to be practiced. Massive transfusion cannot be performed in patients with shock. Emergency surgery could be performed if the operating rooms were not occupied.

### Data

Anonymous and unconsolidated data on road traffic-injured patients were obtained from the trauma registry at Mittaphab Hospital, which was established in 2018. The trauma registry consisted of prehospital records used in the ambulance and medical records used by the hospital. The prehospital records were filled by rescue teams concurrently with patient care, and included the basic patient information and data on the time course, vital signs, injuries, and treatment. The hospital staff was responsible for collecting and registering the data. The data were primarily recorded in the paper-based form and then registered electronically.

The following data were collected: age and sex; time of ambulance dispatch, arrival at the scene, leaving the scene, and arrival at the hospital; name of the rescue team; type of vehicle used by the patient and by the person collided with; prehospital and in-hospital vital signs (heart rate, systolic blood pressure, consciousness level based on the Glasgow Coma Scale [GCS]) measured at the scene and hospital arrival, respectively; injured body part (head, face, neck, chest, abdomen, spine, upper limbs, and lower limbs); outcome (discharged to home, transferred to another hospital, and died); and date of discharge, transfer, or death.

### Measures

The exposure and outcome variables were prehospital time (time interval from ambulance dispatch to arrival at the hospital) and 24-h mortality. The patients who were discharged or transferred to another hospital within 24 h were considered as survivors. The patients’ condition was determined as mild or non-mild based on their prehospital SI and GCS scores, as shown in Table 1. To measure the anatomical severity, the number of seriously injured body parts with more than three points on the Abbreviated Injury Scale (AIS) were determined. However, the Injury Severity Score was not calculated as the six-point AIS coding could not be performed.

**Table 1:** Severity of condition based on the prehospital shock index and Glasgow Coma Scale score

	Prehospital Glasgow Coma Scale score^ac^	
	Less than 13	13 or more
Prehospital shock index^ab^		
1 or more	Non-mild	Non-mild
Less than 1	Non-mild	Mild

^a^Based on the vital signs measured at the scene

^b^Shock index was calculated by dividing the heart rate by the systolic blood pressure

^c^Glasgow Coma Scale score range from 3 to 15

### Analysis

The analysis focused on patients with non-mild conditions for the reasons stated earlier. Prior to the analysis, the patients whose outcomes were unknown were excluded, and any missing values of the variables were supplemented using the multiple imputation method, which included the response time (from ambulance dispatch to arrival at the scene), on-scene time (from arrival at the scene to leaving the scene), transport time (from leaving the scene to arrival at the hospital), and prehospital systolic blood pressure, heart rate, and the GCS score. Considering the amount of missing data, 50 pseudo-complete datasets were created through imputation [34]. Subsequently, the characteristics of the patients with non-mild conditions were compared between those who died within 24 h after the crash and those who survived. This comparison was made using the Wilcoxon rank sum test, Kruskal–Wallis test, or Pearson’s chi-square test, depending on the type and distribution of the variable. Rubin’s rules were used to average across the imputed datasets.

Finally, the relationship between a prehospital time of <60 min and 24-h mortality was examined. First, the propensity score for a prehospital time of <60 min was calculated because the probability of patients having a prehospital time of <60 min might depend on their condition at the scene, which might confound the relationship. To calculate the propensity score, a logistic regression analysis was conducted with adjustment for covariates, including age, sex, rescue team, prehospital SI and GCS scores, serious injury to each body part, time zone when transported, and type of vehicle used by the patients and by the person collided with (Supplementary Table 1). Subsequently, to estimate the association of a prehospital time of <60 min and 24-h mortality, a generalized estimating equation model was used incorporating the inverse probability weights based on the propensity score [35]; the odds ratio (OR) and its 95% confidence interval (CI) were provided. The analysis was performed using SPSS version 27.0 (IBM Corp., Armonk, NY, USA).

## Results

A total of 18,995 patients involved in road traffic crashes visited the Mittaphab Hospital during the study period and 5,604 patients of them were transported by ambulance. Of these, 1,455 patients were transported from other hospitals, 18 patients experienced CPA at the scene, and 8 patients had undocumented outcomes. These patients were excluded from the analysis. A total of 4,123 patients were included in the analyses for missing data imputation. Supplementary Table 2 shows the patient characteristics before the imputation. Additionally, the patients were classified into those with mild or non-mild conditions based on the prehospital SI and GCS scores.

Table 2 shows the characteristics of 701 patients with non-mild conditions, including those who died within 24 h (*n* = 35) and those who survived (*n* = 666). Of the patients with a median age of 25 years, 73% were men, 91% were driving or riding 2- or 3-wheel motor vehicles during the crash, and 44% were transported by Vientiane Rescue 1623, the first rescue team established in Laos with the largest number of members and equipment. The median response time, on-scene time, and transport time were 12, 7, and 16 min, respectively, and 475 patients had a prehospital time of less than 60 min. The most common injured parts were the head (30%), face (26%), and lower limbs (22%). Compared with those who survived, patients who died were more likely to have injuries in the head, chest, and abdomen.

**Table 2:** Characteristics of traffic-injured patients with non-mild conditions who were transported to Mittaphab Hospital by ambulance after a road traffic crash from May 2018 to April 2019 and the 24-h mortality rates^a^

	Died (*n* = 35)	Survived (*n* = 666)
Age (years)	23 [19, 39]	25 [21, 35]
Sex (male)	26 (74%)	489 (73%)
Type of vehicle used by the patients		
Pedestrian	0 (0%)	22 (3.3%)
Bicycle	0 (0%)	4 (0.6%)
2- or 3-wheel	33 (94%)	606 (91%)
4-wheel	2 (5.7%)	32 (4.8%)
Rescue team (Vientiane Rescue 1623)	15 (45%)	293 (44%)
Prehospital time		
Response time^b^	10 [5, 32]	11 [5, 26]
On-scene time^c^	7 [5, 9]	8 [5, 13]
Transport time^d^	13 [9, 35]	20 [10, 37]
Prehospital time (less than 60 min)	22 (63%)	453 (68%)
Prehospital vital signs^e^		
Shock index	0.8 [0.7, 0.9]	0.8 [0.7, 1.0]
Glasgow Coma Scale	3 [3, 3]	6 [5, 7]
In-hospital vital signs^f^		
Shock index	0.9 [0.5 1.3]	0.8 [0.7, 0.9]
Glasgow Coma Scale	3 [3, 4]	12 [7, 15]
Serious injury to each body part^g^		
Head	32 (91%)	175 (26%)
Face	21 (60%)	158 (24%)
Neck	7 (20%)	5 (0.8%)
Chest	17 (49%)	29 (4.4%)
Abdomen	12 (34%)	26 (3.9%)
Spine	0 (0%)	2 (0.3%)
Upper limbs	4 (11%)	67 (10%)
Lower limbs	13 (37%)	140 (21%)
Number of seriously injured body parts ^h^	3 [2, 4]	2 [1, 2]

^a^Values other than the frequency (%) are expressed as median [interquartile range]

^b^From ambulance dispatch to arrival at the scene

^c^From arrival at the scene to leaving the scene

^d^From leaving the scene to hospital arrival

^e^Measured at the scene

^f^Measured at hospital arrival

^g^Injury with more than three points on the Abbreviated Injury Scale (AIS)

^h^Number of body parts containing injuries with more than three points on the AIS

Table 3 shows the association between the prehospital time and 24-h mortality. The proportion of 24-h mortality was 4.7% and 5.4% in patients with a prehospital time of <60 min and those with a prehospital time of 60 min or longer, respectively. No significant association was found between the prehospital time and 24-h mortality.

**Table 3:** Proportion of 24-h mortality by prehospital time among traffic-injured patients with non-mild condition who were transported to Mittaphab Hospital by ambulance after a road traffic crash from May 2018 to April 2019, and the association of prehospital time with 24-h mortality

Prehospital time^a^	%	OR (95% CI)^b^
Less than 60 min	4.7	0.99 (0.42–2.42)
60 min or more	5.4	Reference

*OR* odds ratio, *CI* confidence interval

^a^Sum of response time, on-scene time, and transport time

^b^Estimated using the generalized estimating equations model incorporating the inverse probability weights using the propensity score for prehospital time of <60 min (based on age, sex, rescue team, prehospital shock index, prehospital Glasgow Coma Scale score, serious injury to each body part with more than three points on the Abbreviated Injury Scale, time zone when transported, and the type of vehicle used by the patients and by the person collided with)

## Discussion

A prehospital time of <60 min was not significantly associated with the 24-h mortality in road traffic-injured patients with non-mild conditions in Laos. To interpret this finding, caution should be exercised because the impact of prehospital time was not precisely estimated due to a small number of outcomes, though potential explanations for the lack of significant association were provided below for future research.

First, hospitals do not have the capacity to provide high-quality resuscitation, even if the patients are transported to the hospital within an hour. The quality of in-hospital care provided to trauma patients is not only determined solely by the skills of the surgeons, but also by the diagnostic skills, supportive treatment, and perioperative management. To stabilize shock patients with active bleeding, massive transfusions are required [36]. Even when operations are perfectly conducted, the outcomes depend highly on the quality of anesthetic management and postoperative care provided in the intensive care unit. Such in-hospital care is not always available in Laos.

Second, even if the time from the injury to hospital arrival was shortened, the time from hospital arrival to resuscitation might not have been shortened. In Laos, the prehospital information is not shared with the hospital before hospital arrival, which delays the formation of trauma teams to resuscitate the patients. Moreover, it is not always possible to conduct emergency operations because of the limited capacity to manage operating rooms. In this case, the patients needed to wait until the ongoing operations were completed, which may be deleterious, especially for patients requiring urgent care.

Third, traffic crashes that occur in suburban areas may be less likely witnessed than those that occur in urban areas. In addition, the emergency call numbers may not be widely known and are less likely to be known by people living in suburban areas, as EMS is not officially provided as a public service. As a result, some emergency calls and ambulance dispatches may be delayed. If this was the case, paradoxically, the outcomes of patients transported from suburban areas with a longer prehospital time could be better than those from urban areas as patients requiring urgent care were more likely to die at the scene before they were noticed in suburban areas, and only those with better condition were transported to the hospital.

This study had four major limitations. First, there was an inherent uncertainty in our estimates due to the large missing data with a small number of outcomes despite the data imputation. In order to deal with the missing data, a large number of imputed datasets were generated for analysis. To consider a large number of potential confounders relative to a small number of outcomes, the propensity score method was used. Yet, the impact of prehospital time should be further precisely estimated in a larger sample size. Second, there might be a potential misclassification of non-mild conditions into mild in patients with severe head or torso injuries, if their prehospital SI and GCS scores indicated mild conditions despite sustaining severe injuries. These cases were not included in the analysis. This might have led to the underestimation of the relationship between prehospital time and mortality. However, this could be occurred only if there were many such cases and the prehospital time was shortened due to the presence of head or torso injuries. Third, there was a paucity of information, such as the time from the crash to ambulance dispatch and the location of the crash, which might help explain the lack of association. Fourth, our findings are not applicable to other low-resource settings that employ a “stay-and-play” type of EMS, unlike the “scoop-and-run” type, which exists in Laos. In the “stay-and-play” type, on-scene treatment and resuscitation, such as endotracheal intubation and blood transfusion at the scene, are prioritized over rapid transportation [37–39].

In conclusion, the prehospital time was not significantly associated with 24-h mortality among traffic-injured patients with non-mild conditions, transported by ambulance in Laos. The quality of prehospital and in-hospital care that might affect the survival should be evaluated further in a larger sample size to estimate the impact of prehospital time more precisely.

## Supplementary Information

Below is the link to the electronic supplementary material.

Supplementary file1 (DOCX 43 kb)
