# Supplementary material for: Relationship Between Prehospital Time and 24-h Mortality in Road Traffic-Injured Patients in Laos
Source: World J Surg. 2022 Jan 18;46(4):800–6. doi: 10.1007/s00268-022-06445-9 (PMC8885552; doi:10.1007/s00268-022-06445-9)
Supplement: Supplementary file 1 — Supplementary file1 (DOCX 43 kb) [file 268_2022_6445_MOESM1_ESM.docx]

Electronic Supplementary Material

Article title

Relationship Between Prehospital Time and 24-h Mortality in Road Traffic-Injured Patients in Laos

Journal name

World Journal of Surgery

Author names

Takaaki Suzuki ^1, 2^, Oulaivanh Phonesavanh ^3^, Snong Thongsna ^4^, Yoshiaki Inoue ^2^, and Masao Ichikawa ^1^

Affiliation

^1^ Department of Global Public Health, Faculty of Medicine, University of Tsukuba, 1-1-1 Tennodai, Tsukuba, Ibaraki, Japan 305-8575

^2^ Department of Emergency and Critical Care Medicine, Faculty of Medicine, University of Tsukuba, 1-1-1 Tennodai, Tsukuba, Ibaraki, Japan 305-8575

^3^ Emergency Department, Mittaphab Hospital, Ministry of Health, Vientiane, Lao People’s Democratic Republic

^4^ Ministry of Health, Vientiane, Lao People’s Democratic Republic

Corresponding author

Takaaki Suzuki

University of Tsukuba, 1-1-1 Tennodai, Tsukuba, Ibaraki, Japan 305-8575

Email: [takasuzuki@md.tsukuba.ac.jp](mailto:takasuzuki@md.tsukuba.ac.jp)

Supplementary Table 1: Propensity Score Calculation: Derived from 16 Prehospital Characteristics

|  | | **B** | **SE** | **Sig** | **Exp(B)** | **95%CI of Exp(B)** | |
| --- | --- | --- | --- | --- | --- | --- | --- |
|  |  |  |  |  |  | **Lower** | **Upper** |
| Age (years) | | .000 | .003 | .910 | 1.000 | .994 | 1.006 |
| Sex (male) | | -.142 | .086 | 099 | .868 | .733 | 1.027 |
| Rescue team  (Vientiane Rescue 1623) | | .454 | .081 | <.001 | 1.574 | 1.343 | 1.844 |
| Prehospital  vital signs ^a^ | Shock index | -.219 | .351 | .534 | .804 | .403 | 1.604 |
|  | Glasgow Coma Scale | -.017 | .021 | .418 | .983 | .943 | 1.025 |
| Presence of serious injury in each body part ^b^ | Head | -.357 | .176 | .042 | .700 | .496 | .988 |
|  | Face | -1.232 | .710 | .083 | .292 | .072 | 1.177 |
|  | Neck | -.111 | .156 | .479 | .895 | .659 | 1.216 |
|  | Chest | .314 | .330 | .342 | 1.369 | .716 | 2.616 |
|  | Abdomen | -.121 | .316 | .701 | .886 | .477 | 1.646 |
|  | Spine | -.464 | .599 | .438 | .629 | .194 | 2.036 |
|  | Upper limbs | -.027 | .143 | .851 | .973 | .735 | 1.290 |
|  | Lower limbs | -.236 | .104 | .023 | .790 | .644 | .969 |
| Time zone when transported ^c^ | | -.124 | .086 | .205 | .883 | .729 | 1.070 |
| Type of vehicle | Used by the patients ^d^ | -.153 | .081 | .060 | .858 | .732 | 1.007 |
|  | Used by the person collided with ^e^ | .052 | .033 | .112 | 1.053 | .988 | 1.123 |
| Constant | | 1.406 | .466 | .003 | 4.078 | 1.634 | 10.179 |

B, beta; SE, standard error; Sig, significance; Exp(B), exponential of beta; CI, confidential interval

^a^ Measured at the scene.

^b^ Serious injury was injury with more than three points on the Abbreviated Injury Scale.

^c^ Time zone was divided into daytime (8AM-16PM) and night time (16PM-8AM).

^d^ Type of vehicle used by the patients was either pedestrian, bicycle, 2- or 3-wheel or 4-wheel.

^e^ Type of vehicle used by the person collided with was either pedestrian, bicycle, 2- or 3-wheel, 4-wheel or none (meaning single vehicle collision).

Supplementary Table 2: Characteristics of 4,123 traffic-injured patients who were transported to Mittaphab Hospital by ambulance after a road traffic crash from May 2018 to April 2019 and the missing values ^a^

|  | | **Total (n=4,123)** | **% missing** |
| --- | --- | --- | --- |
| Age (years) | | 25 [20, 35] | 1 (0%) |
| Sex (male) | | 2,774 (67%) | 0 (0%) |
| Type of vehicles used by the patients | Pedestrian | 164 (4.0%) | 8 (0.2%) |
|  | Bicycle | 30 (0.7%) |  |
|  | 2- or 3-wheel | 3,735 (91%) |  |
|  | 4-wheel | 186 (4.5%) |  |
| Rescue Team (Vientiane Rescue 1623) | | 1,976 (48%) | 5 (0.1%) |
| Prehospital time | Response time ^b^ | 9 [5, 14] | 1,312 (32%) |
|  | On-scene time ^c^ | 6 [5, 20] | 1,094 (27%) |
|  | Transport time ^d^ | 14 [9, 20] | 1,011 (25%) |
| Prehospital  vital signs ^e^ | Shock index | 0.8 [0.7, 0.8] | 2,451 (59%) |
|  | Glasgow Coma Scale | 15 [15, 15] | 94 (2.3%) |
| In-hospital  vital signs ^f^ | Shock index | 0.7 [0.7, 0.8] | 2,562 (52%) |
|  | Glasgow Coma Scale | 15 [15, 15] | 159 (3.9%) |
| Serious injury to each body part ^g^ | Head | 304 (7.4%) | 0 (0%) |
|  | Face | 356 (8.6%) |  |
|  | Neck | 14 (0.3%) |  |
|  | Chest | 92 (2.2%) |  |
|  | Abdomen | 75(1.8%) |  |
|  | Spine | 15 (0.4%) |  |
|  | Upper limbs | 331 (8.0%) |  |
|  | Lower limbs | 697 (17%) |  |
| Number of seriously injured body parts ^h^ | | 0 [0, 1] | 0 (0%) |
| 24-h mortality | | 42 (1.0%) | 0 (0%) |
| In-hospital mortality | | 83 (2.0%) | 0 (0%) |

^a^ Values other than % are expressed as median [interquartile range].

^b^ From ambulance dispatch to arrival at the scene.

^c^ From arrival at the scene to leaving the scene.

^d^ From leaving the scene to hospital arrival.

^e^ Measured at the scene.

^f^ Measured at hospital arrival.

^g^ Injury with more than three points on the Abbreviated Injury Scale (AIS).

^h^ Number of body parts containing injuries with more than three points on the AIS.
